# Intrathecal morphine dose optimization in robotic-assisted laparoscopic hysterectomy: a dual-center cohort study

**DOI:** 10.1007/s11701-026-03445-y

**Published:** 2026-05-12

**Authors:** Andrea Russo, Federica Perelli, Paola Aceto, Martina Arcieri, Federica Bernardini, Sara Pregnolato, Rossana Moroni, Teresa Dogareschi, Francesco Meroi, Barbara Costantini, Valerio Gallotta, Francesco Fanfani, Lorenza Driul, Anna Fagotti, Tiziana Bove, Stefano Restaino, Massimo Antonelli, Giuseppe Vizzielli

**Affiliations:** 1 Department of Emergency, Anaesthesia, and Intensive Care Medicine, Fondazione Policlinico Universitario A. Gemelli IRCCS, Rome, Italy; 2 Pediatric Gynecology Unit, Meyer Children’s Hospital IRCCS, Florence, Italy; 3 Gynecology and Obstetrics Department, Azienda USL Toscana Centro, Santa Maria Annunziata Hospital, Florence, Italy; 4 Department of Biotechnological Basic Science, Intensive and Perioperative Medicine, Catholic University of the Sacred Heart, Rome, Italy; 5 Clinic of Obstetrics and Gynecology, Santa Maria Della Misericordia University Hospital, Azienda Sanitaria Universitaria Friuli Centrale, Udine, Italy; 6 Gynecologic Oncology Unit, Department of Woman and Child Health and Public Health, Fondazione Policlinico Universitario A. Gemelli IRCCS, Rome, 00168 Italy; 7 Department of Medicine (DMED), University of Udine, Udine, Italy; 8 Department of Emergency, University Hospital of Udine, Azienda Sanitaria Universitaria Friuli Centrale, Santa Maria della Misericordia, Piazzale Santa Maria della Misericordia 15, Udine, 33100 UD Italy; 9 UniCAMILLUS, International Medical University, Rome, Italy; 10 Catholic University of the Sacred Heart, Rome, Italy; 11 PhD School in Biomedical Sciences, Gender Medicine, Child and Women Health, University of Sassari, Sassari, Italy; 12 University Hospital “Santa Maria della Misericordia”, Piazza Santa Maria della Misericordia, 15, Udine, Italy

**Keywords:** Intrathecal morphine, Postoperative pain, Robotic-assisted laparoscopic hysterectomy, VAS scale, Opioid side effects, Anaesthesia management.

## Abstract

**Background:**

Optimized perioperative analgesia is a critical component of Enhanced Recovery After Surgery (ERAS) pathways in robotic-assisted laparoscopic hysterectomy (RALH). In high-volume robotic programs, predictable pain control may influence early mobilization, postoperative stability, and discharge planning. This study evaluated the analgesic efficacy and safety of two low-dose intrathecal morphine (ITM) regimens (0.10 mg vs. 0.15 mg) in patients undergoing RALH.

**Methods:**

We conducted a retrospective dual-center cohort study including 100 women who received spinal anesthesia with 0.10–0.15 mg of preservative-free intrathecal morphine, with or without levobupivacaine, prior to general anesthesia for RALH. Postoperative pain was assessed using the Visual Analog Scale (VAS) at three time points (PACU arrival, PACU discharge, and 24 h postoperatively). Rescue opioid use, hemodynamic events, postoperative nausea and vomiting (PONV), pruritus, and recovery parameters (Alderete Score) were recorded. Comparative analyses were performed between the two ITM dose groups.

**Results:**

Pain scores remained consistently low across all time points (median VAS = 0; *p* = 0.302), with rescue analgesia required in 7% of patients (*n* = 7/100). Compared with the 0.10 mg group, the 0.15 mg group demonstrated significantly lower pain scores and reduced supplemental opioid requirements. Higher rates of pruritus, PONV, and hypotensive episodes were observed in the 0.10 mg group. No cases of respiratory depression or prolonged PACU stay were recorded. Median Alderete Scores were consistently optimal (10/10), indicating stable postoperative recovery.

**Conclusion:**

Low-dose intrathecal morphine provides effective, opioid-sparing, and motor-preserving analgesia in robotic-assisted laparoscopic hysterectomy. In this cohort, the 0.15 mg regimen was associated with improved analgesic balance without an increase in clinically significant adverse events. Within ERAS-based robotic pathways, optimized intrathecal morphine dosing may support predictable recovery and perioperative stability. Observational design precludes causal inference. Prospective randomized studies are warranted to confirm these findings.

**Trial registration:**

The Ethics Committee approved the study (Protocol ID 3307/2020) on July 6th, 2020, and it was registered in clinicaltrial.gov (NCT07169604).

## Introduction

Postoperative pain management is crucial for improving functional recovery, reducing complications, and enhancing patient-reported outcomes following major gynecologic surgery [1–4]. In the context of robotic-assisted laparoscopic hysterectomy (RALH), which is increasingly performed for both oncologic and benign reasons, the implementation of ERAS protocols has revolutionized perioperative care [5, 6]. These protocols emphasize multimodal, opioid-sparing pain management, early mobilization, and reduced hospital stays [7, 8]. Despite advances in minimally invasive techniques, postoperative pain in RALH remains significant and is sometimes inadequately managed. Traditional systemic opioids, although effective, carry a range of side effects—nausea, vomiting, ileus, sedation, pruritus—and can lead to delayed discharge and a higher risk of long-term opioid use [9]. Epidural analgesia, although regarded as the gold standard for open surgery, has limitations in laparoscopic cases, including technical complexity, motor blockade, urinary retention, and the potential for epidural hematoma [10, 11]. Intrathecal morphine (ITM), a long-acting lipophilic opioid administered as a single spinal dose, offers significant advantages in terms of efficacy, simplicity, and cost-effectiveness [12–15]. ITM provides up to 24 h of analgesia, with a low systemic burden and minimal interference with motor function [16]. Its value in urologic and orthopedic surgery is well established, and emerging evidence supports its role in gynecologic laparoscopy [7, 12]. Nevertheless, limited data exist regarding its use in real-world RALH within ERAS frameworks, and even fewer studies have explored its applicability to same-day discharge protocols [13, 14]. Furthermore, the impact of ITM in high-risk populations (e.g., obese or hypertensive patients) remains under-investigated. This study aimed to evaluate the analgesic efficacy, safety profile, and recovery outcomes associated with intrathecal morphine—administered alone or with levobupivacaine—in patients undergoing RALH. We specifically analyzed pain trajectories, need for rescue analgesia, opioid-related side effects, and readiness for discharge as reflected by Alderete Scores.

## Materials and methods

**Study design and patients**: We conducted an observational, retrospective, dual-center cohort study including 100 women who underwent RALH at the Fondazione Policlinico Universitario A. Gemelli IRCCS in Rome, Italy, and the University Hospital “Santa Maria della Misericordia” in Udine, Italy, between January 2021 and December 2024. Ethical approval for the study was obtained from the Ethics Committee of Fondazione Policlinico Universitario Agostino Gemelli IRCCS (Protocol ID: 3307/2020) on 6 July 2020 and was registered at ClinicalTrials.gov (NCT07169604). The study was conducted in accordance with the principles of the Declaration of Helsinki. Written informed consent to participate was obtained from all participants prior to inclusion in the study. All procedures were conducted in accordance with institutional ERAS protocols. Eligible participants were adult women (≥ 18 years) who received spinal anesthesia with 0.10–0.15 mg of preservative-free intrathecal morphine, administered with or without 1 mL of 0.75% levobupivacaine prior to the induction of general anesthesia. Dose selection rationale and adjunct intrathecal levobupivacaine Intrathecal morphine dosing (0.10 mg or 0.15 mg preservative free) and the decision to add 1 mL of 0.75% levobupivacaine were chosen by the attending anaesthesiologist according to institutional practice and individual patient characteristics (age, comorbidities, prior opioid exposure, perceived surgical complexity). These two low doses were selected based on prior literature supporting analgesic efficacy with an acceptable safety profile in laparoscopic and ambulatory gynaecologic surgery and on local practice patterns favoring minimal motor block while prolonging analgesia. To transparently report real world practice, levobupivacaine use and ITM dose are presented as administered; no protocolized randomization determined allocation. Counts of patients receiving levobupivacaine are provided in Table 1. Patients were scheduled for RALH for benign reasons (e.g., symptomatic fibroids, adenomyosis) or malignant/pre-malignant conditions (e.g., endometrial cancer, atypical hyperplasia). Exclusion criteria were contraindications to neuraxial anesthesia (such as coagulopathies, platelet count below 100,000/mm³ or thrombocytopathy), severe valvular heart disease, left ventricular ejection fraction less than 35%, history of obstructive or restrictive pulmonary disease, or significant neurological disorders. **Anesthesia and analgesia protocol**: A 25-gauge Whitacre spinal needle was inserted at the L 3 – L 4 interspace under aseptic conditions, with ultrasound guidance used in obese patients (body mass index (BMI) over 35 kg/m²). The choice of intrathecal morphine dose and the use of levobupivacaine were left to the discretion of the attending anesthesiologist, according to clinical judgment and institutional practice. Intraoperative monitoring included standard hemodynamics and capnography. Pain and recovery were assessed using a 10-cm Visual Analog Scale (VAS), where 0 indicated “no pain” and 10 represented “the worst imaginable pain”. Assessments were performed by trained nursing staff in the PACU and surgical ward, and all patients received standardized preoperative instructions on how to use this score. In total, two dedicated PACU nurses and two dedicated ward nurses per center conducted VAS and Aldrete scoring according to a common institutional protocol. Evaluators received standardized training on administration of the 10 cm Visual Analog Scale and on Aldrete scoring prior to study initiation. Patients were provided with uniform preoperative instructions on how to rate pain using the VAS. Evaluators were aware of the anaesthetic technique (non blinded), but use of standardized assessment forms and prespecified time points (T0: PACU arrival; T1: after PACU discharge; T2: 24 h postoperatively) aimed to reduce measurement variability. All patients received postoperative regular IV paracetamol (1 g every 8 h). Postoperative Tramadol 100 mg IV was administered as rescue analgesia if the VAS score was 5 or higher. Metoclopramide 10 mg was intravenously administered before extubation to prevent postoperative nausea and vomiting. Furthermore, Ondansetron 4 mg was used as postoperative rescue therapy. Measurements were recorded at three time points: **.

**Table 1:** Main clinical and demographic characteristics of the sample (*N* = 100)

Surgical pathology	Freq.	%
*Malignant and precancerous pathology*	67	67.0
*Benign pathology*	33	33.0
*missing*	0	0
Comorbidity		
*Yes*	89	89.0
*No*	11	11.0
Comorbidity details (*n* = 89)		
*Obesity And Arterial Hypertension*	24	27.0
*Obesity*,* Arterial Hypertension And Other*	21	23.6
*Obesity*	12	13.5
*Arterial Hypertension*	10	11.2
*Obesity And Other*	9	10.1
*Arterial Hypertension*,* Other*	7	7.9
*Other*	6	6.7
Post-operative complications		
*No*	100	100.0
ASA		
*II*	67	67.0
*III*	33	33.0
Levobupivacaine use		
	95	95.0
	**(mean ± SD)**	
Duration of surgery in minutes	166.3 ± 33.7	
Diuresis (mL)	354.8 ± 200.6	
Total amount of fluids (mL)	1256.5 ± 304.7	
Hospital length of stay (hours)	62.6 ± 25.5

- T0: immediately post-surgery in the PACU,
- T1: after PACU discharge,
- T2: 24 h post-surgery.

Additional data included: need for postoperative rescue analgesics (Tramadol 100 mg iv), incidence of pruritus, PONV, intraoperative and postoperative arterial hypotension (mean arterial pressure (MAP) < 65 mmHg), use of vasoactive medications (Etilephrine), and recovery as assessed by the Alderete Score (range 0–10). **Statistical analysis**: The sample has been described in its clinical and demographic characteristics applying descriptive statistics techniques. Number of patients (n), mean, standard deviation (SD), median (first and third quartile, Q1-Q3), minimum and maximum were presented for VAS and Alderete score (continuous variables); for all the other continuous variables only mean and standard deviation were presented. For categorical variables the absolute(n) and percentage (%) frequency were presented for each category. A repeated measures analysis of variance (RM-ANOVA) was conducted to examine the effects of intrathecal morphine dose (0.10 mg vs. 0.15 mg) on pain scores over time. The model included morphine dose as a between-subject factor and time as a within-subject factor, allowing evaluation of main effects of group, time, and their interaction.

Comparative analyses were conducted between morphine 0,10 and morphine 0,15 groups to evaluate differences in opioid consumption, hypotensive episodes, and adverse effects (pruritus and PONV). Mann-Whitney U test was used for continuous variables, while Fisher’s exact test and Chi-square test were employed for categorical variables as appropriate.

Comparisons of pain scores across time points were conducted using the Friedman test, as the data were non-normally distributed. Statistical significance was defined as *p* < 0.05. Analyses were performed using R version 4.3.0.

## Results

A total of 100 patients were included. The mean age was 49.7 years (± 8.4), and the mean BMI was 36.4 kg/m² (± 7.9), with values ranging from 23 to 67. 67 patients underwent RALH for malignant or pre-malignant indications, while the remaining 33 had benign conditions. Of the 100 patients, 23 received 0.10 mg and 77 received 0.15 mg of intrathecal morphine. Levobupivacaine was not administered in 3 patients in the 0.10 mg group and in 2 patients in the 0.15 mg group. The overall distribution of levobupivacaine use was similar between groups. Comorbidities were present in 89% of the cohort, particularly obesity (BMI ≥ 30), arterial hypertension, or both (Table 1).

## Postoperative pain

**Fig. 1:**
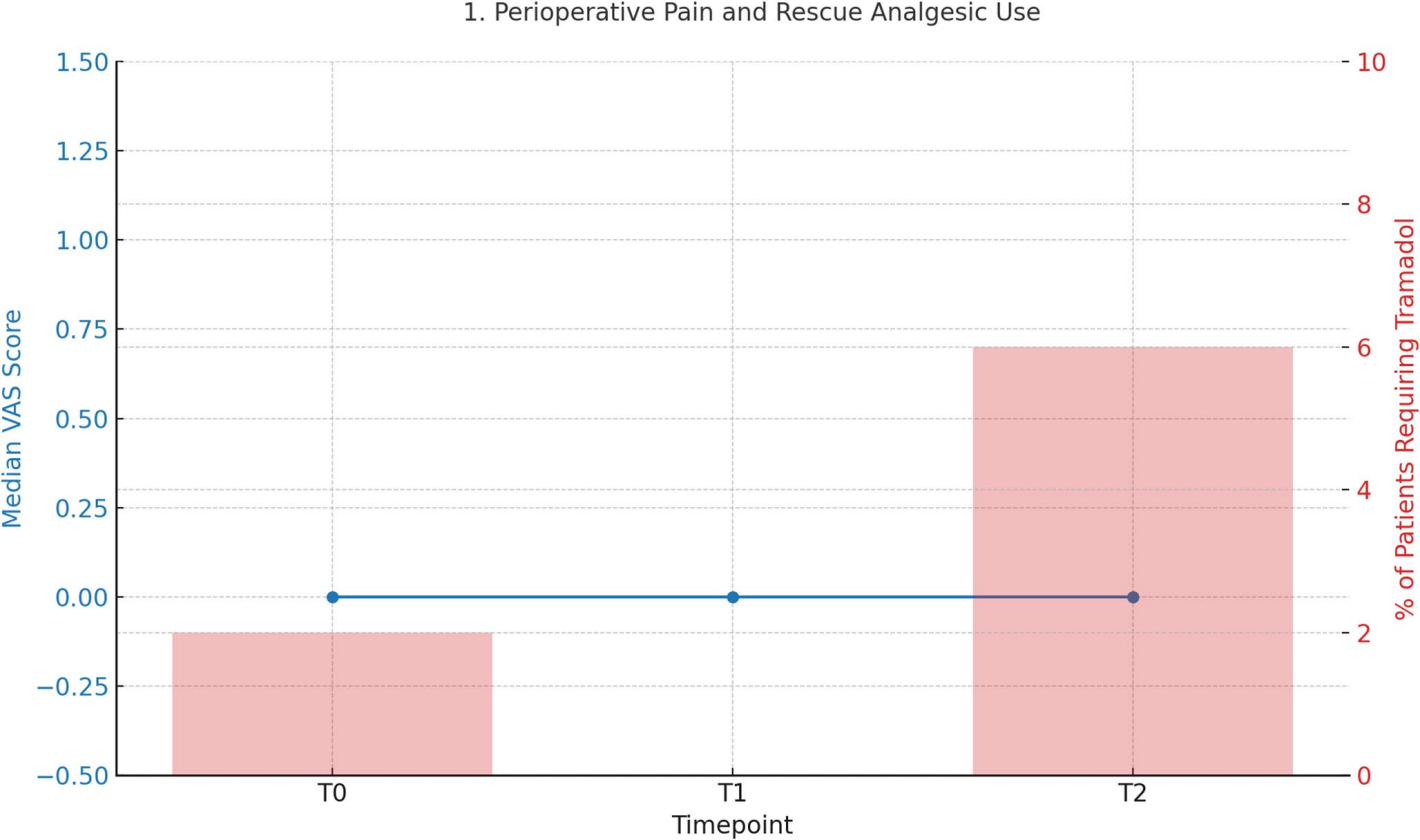
Perioperative pain and rescue analgesic use

Postoperative pain remained consistently low at all assessed time points (Fig. 1). Mean VAS scores were 0.9 at T0, 0.7 at T1, and 1.0 at T2, with a median value of zero across all assessments. The Friedman test revealed no statistically significant differences in VAS scores over time (*p* = 0.302), confirming stable and adequate analgesia (Table 2). Rescue analgesia with tramadol was required in only seven patients (7%): three during PACU recovery and four on postoperative day 1 (Table 3). Stratification according to levobupivacaine use did not demonstrate statistically significant differences. Notably, all patients who received tramadol had low VAS scores at the time of administration, suggesting that in some cases rescue analgesia was administered in a precautionary rather than a clinically mandatory fashion. Tables 4, 5.

**Table 2:** VAS main descriptive statistics in T0, T1 and T2

	*N*	Min	Max	Mean	SD.	Median	Q1	Q3
VAS T0	100	0	5	0.24	0.9	0	0	0
VAS T1	100	0	4	0.15	0.7	0	0	0
VAS T2	99	0	6	0.32	1.0	0	0	0

**Table 3:** Descriptive statistics of VAS value by use of Tramadol (*N* = 100)

			VAS T0	VAS T1	VAS T2
**Use of tramadol in recovery**	No	N	97	97	97.0
		mean	0.15	0.14	0.31
		SD	0.68	0.66	0.97
		Min	0.0	0.0	0.0
		Max	4.0	4.0	6.0
		Median	0.0	0.0	0.0
	Yes	N	3.0	3.0	3.0
		mean	3.0	2.0	0.67
		SD	2.65	2.65	1.15
		Min	0	0.0	0.0
		Max	5.0	5.0	2.0
		Median	4	1	0
**Use of tramadol in Post Operative Day 1**	No	N	93.0	93.0	93.0
		mean	0.22	0.22	0.32
		SD	0.86	0.85	0.99
		Min	0.0	0.0	0.0
		Max	5.0	5.0	6.0
		Median	0.0	0.0	0.0
	Yes	N	7.0	7.0	7.0
		mean	0.57	0.0	0.29
		SD	1.51	0.0	0.76
		Min	0.0	0.0	0.0
		Max	4.0	0.0	2.0
		Median	0.0	0.0	0.0

**Table 4:** Distribution of PONV events and ondansetron usage

	*N*	%
Post Operative Nausea and Vomiting and use of Ondansetron		
PONV in T2	3	3.0
Ondansetron in recovery	2	2.0
PONV in T2, Ondansetron in day 1	1	1.0
PONV in T1 and T2, Ondansetron in day 1	1	1.0
Ondansetron in day 1	1	1.0
No PONV, no Ondansetron	92	92.0
Total	100	100.0

**Table 5:** PONV status for patients receiving ondansetron at recovery or POD 1

		PONV T0	PONV T1	PONV T2
Use of ondansetron in recovery				
No	98			
Yes	2	0	0	0
0	0	Missing		
Use of ondansetron in Post Operative Day 1				
No	97			
Yes	3	0	1	1
0	0	1		
0	0	0		

## Comparisons between morphine 0,10 and morphine 0,15 group

Results of the repeated measures ANOVA demonstrated a significant main effect of group (0.10 mg vs. 0.15 mg; *p* = 0.039). The morphine 0,15 group demonstrated significantly lower pain scores compared to the morphine 0,10 group, with a mean difference of 1.05 points lower pain intensity. No significant main effect of time was found (*p* > 0.05), indicating that pain scores did not change significantly across the measurement periods when considering both groups together. The analysis revealed no significant group × time interaction (all p-values > 0.05). This indicates that both morphine groups exhibited similar patterns of change over time, with no differential temporal effects between the treatment conditions. Additional opioid dosing beyond the primary morphine treatment did not demonstrate a measurable impact on pain outcomes. Morphine 0,10 group: Pain scores remained relatively stable across time points (1.05 → 0.79 → 1.40), showing modest fluctuation around baseline levels. Morphine 0,15 group: Pain scores remained consistently low (≈ 0) across all measurement time points, demonstrating sustained analgesic efficacy.

**Total opioid dose.** A Mann-Whitney U test revealed a statistically significant difference in total opioid consumption between groups (*p* < 0.0001). Patients who received 0.15 mg of intrathecal morphine required significantly less supplemental sufentanil compared with those who received 0.10 mg, indicating that the higher dose provided more effective postoperative pain control.

**Tramadol administration.** During PACU recovery, tramadol was required in 13.0% of patients (3/23) in the morphine 0,10 group, compared with none in the morphine 0,15 group. On postoperative day 1, tramadol was administered to 13.0% (3/23) of patients in the morphine 0,10 group and 5.2% (4/77) in the morphine 0,15 group. These differences were not statistically significant.

**Hypotensive episodes.** Episodes of MAP < 65 mmHg occurred significantly more frequently in the morphine 0,10 group (34.8%) compared with the morphine 0,15 group (10.4%, *p* = 0.009). A borderline significant difference was also noted for systolic arterial pressure < 90 mmHg (*p* = 0.049), again with higher incidence in the morphine 0,10 group.

**Pruritus.** Six patients (26.1%) in the morphine 0,10 group experienced pruritus, whereas no cases were reported in the morphine 0,15 group (*p* < 0.001).

**Postoperative nausea and vomiting (PONV).** Five patients (21.7%) in the morphine 0,10 group reported nausea or vomiting, while no events were observed in the morphine 0,15 group (*p* < 0.001). Similarly, ondansetron was administered to 5 patients (21.7%) in the morphine 0,10 group and to none in the morphine 0,15 group (*p* < 0.001).

**Adverse events.** No cases of respiratory depression, excessive sedation, or other serious opioid-related complications were observed. Overall, hypotension (MAP < 65 mmHg or SAP < 90 mmHg) occurred in 16% of patients, and 20% required transient vasoactive drug support (Table 6). No patients required ICU transfer or prolonged monitoring beyond standard PACU recovery.

**Table 6:** Postoperative adverse events

	*n*	%
Neuromuscular reversal		
No	61	61.0
Yes	39	39.0
Mean Arterial Pressure < 65 mmHg		
No	84	84.0
Yes	16	16.0
Systolic Arterial Pressure < 90 mmHg		
No	84	84.0
Yes	16	16.0
Vasoactive Medications Used		
No	80	80.0
Yes	20	20.0
Pruritus = Yes		
T0	3	3.0
T1	4	4.0
T2	3	3.0

## Recovery metrics

Alderete Scores were uniformly high, with a median of 10 at T0, T1, and T2, suggesting excellent neurologic and physiological recovery (Table 7). No patients experienced delayed arousal, respiratory compromise, or PACU stays longer than 2 h.

**Table 7:** Alderete score

	*N*	Mean	Std.dev.	Min	Max	Median
Alderete score T0	100	9.8	1.0	0	10	10
Alderete score T1	100	9.7	1.4	0	10	10
Alderete score T2	98	10.0	0.0	10	10	10

## Discussion

Our findings demonstrate that low-dose intrathecal morphine (ITM), administered at 0.10–0.15 mg, provides effective and sustained postoperative analgesia in patients undergoing robotic-assisted laparoscopic hysterectomy (RALH). Pain control remained stable throughout the first 24 postoperative hours, with a median VAS score of 0 at all timepoints and a limited requirement for rescue analgesia. These findings are consistent with previous literature supporting the role of ITM as a key component of opioid-sparing strategies within ERAS pathways [1, 5, 7].

In the context of robotic surgery, perioperative optimization extends beyond analgesic efficacy alone. Robotic-assisted procedures are increasingly performed within structured ERAS programs, where predictable recovery trajectories are essential for early mobilization, standardized PACU discharge, and the feasibility of same-day or early discharge protocols [7, 12, 13, 17]. Although workflow metrics were not formally assessed in this study, the uniformly high Alderete Scores and low rescue opioid requirements observed suggest a stable postoperative course compatible with high-efficiency robotic pathways. In high-volume robotic centers, minimizing systemic opioid exposure may contribute to reduced variability in postoperative recovery and more reproducible discharge timelines.

The comparison between the 0.10 mg and 0.15 mg ITM doses represents a clinically relevant aspect of this analysis. Patients receiving 0.15 mg demonstrated significantly lower pain scores and reduced supplemental opioid requirements compared with those receiving 0.10 mg. Interestingly, the lower-dose group exhibited higher rates of pruritus, PONV, and hypotensive episodes. While this may appear counterintuitive, similar observations have been described in studies emphasizing the interaction between inadequate baseline neuraxial analgesia and increased systemic opioid administration [2, 16, 18]. It is plausible that insufficient intrathecal analgesia in the 0.10 mg group resulted in greater systemic opioid use, thereby increasing opioid-related adverse effects such as nausea. From a mechanistic perspective, a slightly higher intrathecal dose may achieve more stable segmental opioid receptor activation during the early inflammatory phase, reducing breakthrough pain and limiting subsequent systemic opioid exposure [15, 19].

Our results align with previous investigations in minimally invasive gynaecologic surgery. Braga et al. [7] reported reduced postoperative opioid consumption following intrathecal morphine in laparoscopic hysterectomy, while Mulier et al. [19] demonstrated enhanced recovery profiles in gynaecologic laparoscopy. However, these studies did not specifically explore dose optimization in robotic hysterectomy. Similarly, Russo et al. [20] highlighted the importance of balancing intrathecal opioid efficacy and safety in robot-assisted laparoscopic prostatectomy, reinforcing the relevance of dose calibration in robotic surgical settings. Even outside robotic contexts, randomized data have confirmed that low-dose intrathecal morphine (i.e. 0,10–0,20 mg) significantly reduces postoperative opioid consumption without a substantial increase in serious adverse events [18, 21]. Meta-analytic evidence further supports that doses below 0.3 mg are associated with manageable rates of nausea and pruritus and do not significantly increase respiratory depression risk [2, 22].

From a technical standpoint, ITM offers advantages particularly relevant to robotic minimally invasive surgery. Unlike epidural analgesia—which requires catheter placement and may delay ambulation—ITM provides prolonged analgesia with minimal motor impairment and without catheter-related management [7, 9, 10]. This single-shot approach is especially valuable in obese and comorbid patients, who represent a substantial proportion of contemporary robotic gynecologic cases, as reflected in our cohort [6, 13]. Preserving lower-limb motor function while ensuring effective analgesia facilitates early mobilization and supports ERAS objectives.

Importantly, no cases of respiratory depression or excessive sedation were observed in our study, consistent with existing safety data on low-dose ITM in surgical populations [2, 18, 22]. The favorable safety profile observed here reinforces the applicability of ITM within well-monitored robotic surgical programs [17, 23–27].

Several limitations must be acknowledged. First, the retrospective design introduces potential selection and information bias. Second, intrathecal morphine dosing was not randomized but left to anesthesiologist discretion, resulting in unequal group sizes and possible confounding by indication. Third, the absence of a control group without ITM limits direct comparison with alternative analgesic strategies such as TAP blocks or systemic-only regimens [16, 24]. Moreover, adjunct intrathecal levobupivacaine was not standardized. Functional recovery parameters—including time to ambulation, bowel recovery, and validated quality-of-recovery scores—were not prospectively collected, limiting assessment of broader ERAS-related endpoints [23, 28, 29]. Finally, the cohort exhibited very low median pain scores (floor effect), which limits the clinical interpretability of small between group differences in VAS. For these reasons, causality cannot be inferred, and these findings should be interpreted as hypothesis-generating.

Despite these limitations, this dual-center experience reflects pragmatic real-world practice in robotic gynaecologic surgery and suggests that optimized intrathecal morphine dosing may enhance perioperative stability within ERAS-based robotic pathways. Prospective randomized controlled trials incorporating functional recovery metrics and workflow-related outcomes are warranted to confirm these observations and to define the optimal role of ITM in standardized robotic hysterectomy programs. These findings should be interpreted with caution, as the retrospective design and lack of randomization preclude definitive conclusions regarding dose superiority.

## Conclusions

In this dual center retrospective cohort study, low dose intrathecal morphine (0.10–0.15 mg) was associated with low postoperative pain scores and infrequent need for rescue systemic opioids after robotic assisted laparoscopic hysterectomy. An observed association between the 0.15 mg regimen and reduced supplemental opioid requirements was present in adjusted and sensitivity analyses; however, small absolute differences, the non randomized allocation of doses, and potential confounding limit causal inference. These results support the feasibility of low dose ITM within ERAS pathways for robotic hysterectomy but should be confirmed in prospective randomized studies with standardized protocols and functional recovery endpoints.

## Data Availability

the datasets generated and/or analyzed during the current study are available from the corresponding author on reasonable request.

## Abbreviations

BMI: Body mass index
ERAS: Enhanced Recovery After Surgery
ITM: Intrathecal morphine
MAP: Mean arterial pressure
PACU: Postoperative anesthetic care unit
PONV: Postoperative nausea and vomiting
RALH: Robotic-assisted laparoscopic hysterectomy
RM: ANOVA-repeated measures analysis of variance
SD: Standard deviation
TAP: Transversus abdominis plane (blocks)
VAS: Visual Analog Scale
